# Supplementary material for: mRNA 3’UTR lengthening by alternative polyadenylation attenuates inflammatory responses and correlates with virulence of Influenza A virus
Source: Nat Commun. 2023 Aug 15;14:4906. doi: 10.1038/s41467-023-40469-6 (PMC10427651; doi:10.1038/s41467-023-40469-6)
Supplement: Supplementary file 1 — Supplementary Information [file 41467_2023_40469_MOESM1_ESM.pdf]

SUPPLEMENTARY FIGURE TEXT

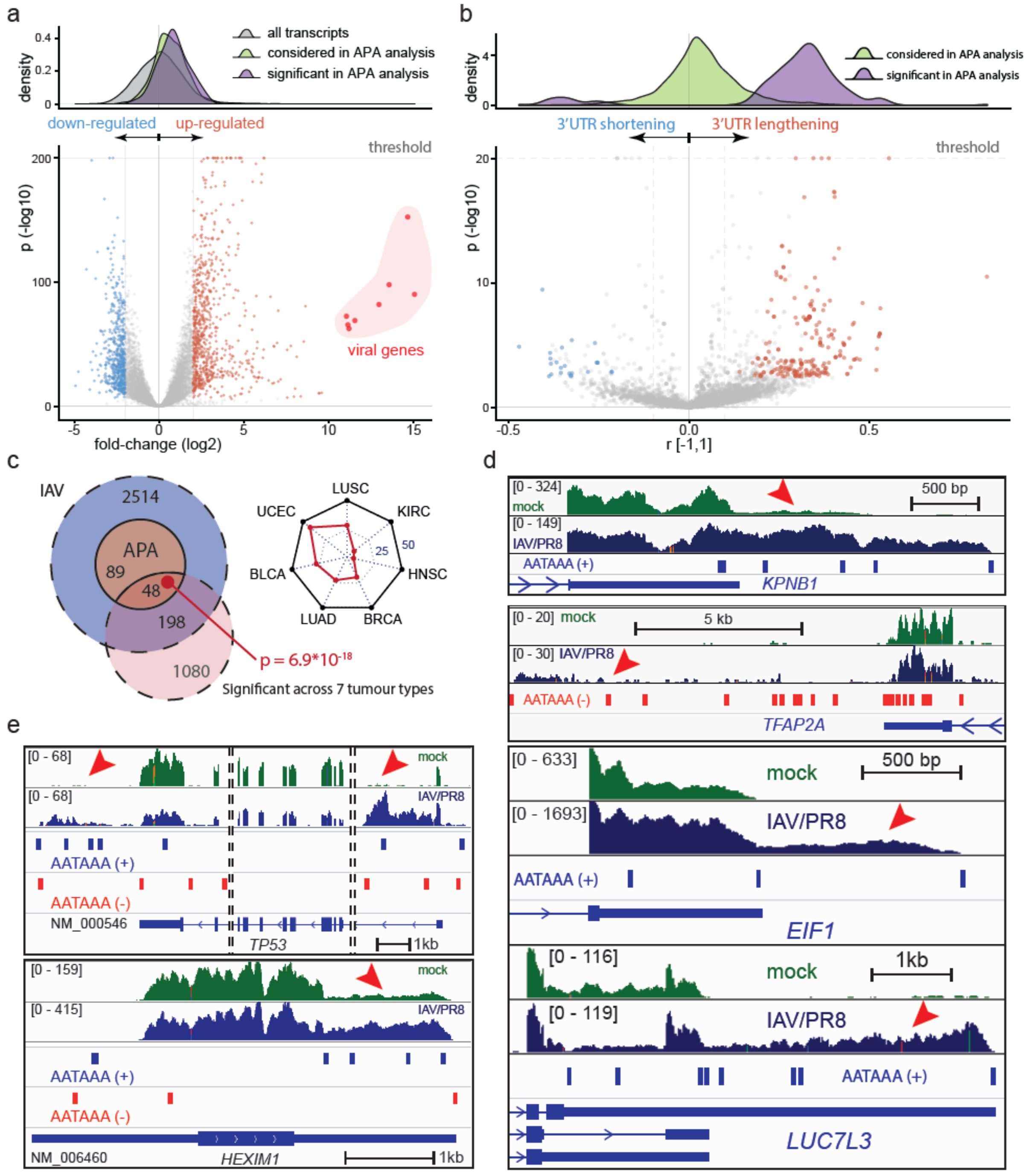

Supplementary Figure 1 | Influenza A causes widespread alternative polyadenylation of host transcripts. (a-e) Related to Fig. 1a. A549 cells were synchronously infected with IAV (strain PR8) at MOI 3 or left uninfected. 24-hours post infection, the cellular RNA was harvested and subjected to RNA sequencing. **(a)** Density (top) and volcano (bottom) plots depict differentially expressed genes between IAV (PR8) and mock-infected cells as annotated (significance thresholds: absolute log2 fold-change > 2, FDR-adjusted p-value < 0.01, further described in the methods section). **(b)** Density (top) and volcano (bottom) plots depicting relations between r- (Pearson product moment correlation coefficient) and p-values in analysis of alternative polyadenylation between IAV (PR8) and mock-infected cells (significance thresholds: FDR-adjusted p-value < 0.05, PD > 0.2, absolute r > 0.1, further described in the methods section). **(c)** Schematic representation of the overlap of genes found to be APA upon IAV (PR8) infection and previously published gene set reported to be APA in different tumor types[28]. Radar

plot further depicts number of overlapping genes found to be APA upon IAV infection and distinct cancer types. Statistics were calculated using one-sided Fisher's exact test. **(d, e)** Read distribution across several exemplar **(d)** and cancer-related **(e)** genes, APA upon IAV infection. Variable 3' UTR regions are highlighted by red arrowheads and canonical polyadenylation sites, encoded at the DNA level as AATAAA, are highlighted by blue or red bars depending on directionality as indicated.

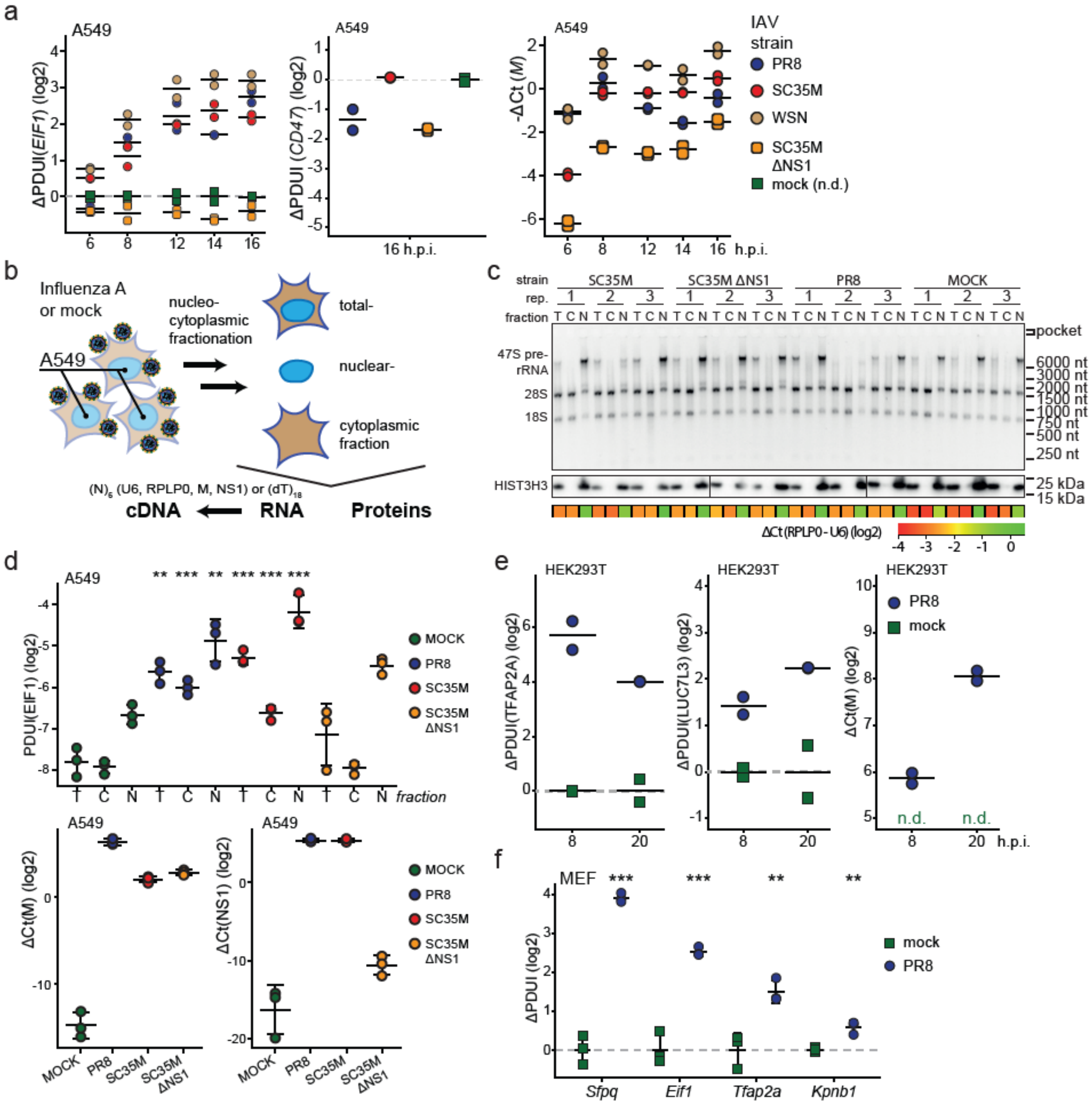

Supplementary Figure 2 | Alternative polyadenylation of host transcripts is mediated by viral protein NS1 irrespective of amino-acids at positions 103/106 and abrogated by G184R mutation. **(a)** Related to Fig. 1c. A549 cells were infected with indicated IAV strains at MOI 3 or left uninfected (mock) and harvested at indicated times. Left: ΔPDUIs of *EIF1* transcript are shown as a measure of APA alongside mean for 2 separately infected wells. Dashed line corresponds to uninfected polyadenylation status. Right: Abundance of viral transcript *M* is shown as a measure of infection kinetics and extent. Middle: CD47 was used as a well characterized control that is not affected by the IAV-induced APA. **(b-d)** Related to Fig. 1d. 3 separate wells of A549 cells were infected with indicated strains of IAV at MOI 3 or left uninfected for 24 hours and then subjected to nucleocytoplasmic fractionation. The presented data is representative of 2 independent repeats. **(b)** Schematic representation of nucleocytoplasmic fractionation, followed by protein and RNA quantification. **(c)** RNA was resolved by agarose gel electrophoresis and stained with Gel Red (Sigma). Depicted abundances of HIST3H3 as nuclear protein and U6 as nuclear RNA were quantified by western blot and RT-qPCR, respectively. **(d)** RNA was reverse transcribed using $(dT)_{18}$ and used for RT-qPCR based quantification of APA of gene EIF1 as well as quantification of expression of viral genes M and NS1, shown alongside mean +/- sd. **(e)** HEK293T cells were infected with IAV (PR8) at MOI 3 or left uninfected for indicated times, followed by RT-qPCR based quantification of APA (*TFAP2A*, *LUC7L3*) and viral transcript abundances (*M*). ΔPDUI as a

measure of APA status is shown alongside mean for 2 separately infected wells. **(f)** Mouse embryonic fibroblasts (MEFs) were infected with IAV (PR8) at MOI 3 or left uninfected for 24 hours, followed by RT-qPCR based quantification of APA (*Sfpq, Eif1, Tfap2a, Kpnb1*). ΔPDUI as a measure of APA status is shown alongside mean +/- sd for 3 separately infected wells. Statistics were calculated using two-sided equal variance t-test. n.s. $p>0.05$, * $p<0.05$, ** $p<0.01$, *** $p<0.001$. ΔCt values were calculated relative to the housekeeping gene *RPLP0*.

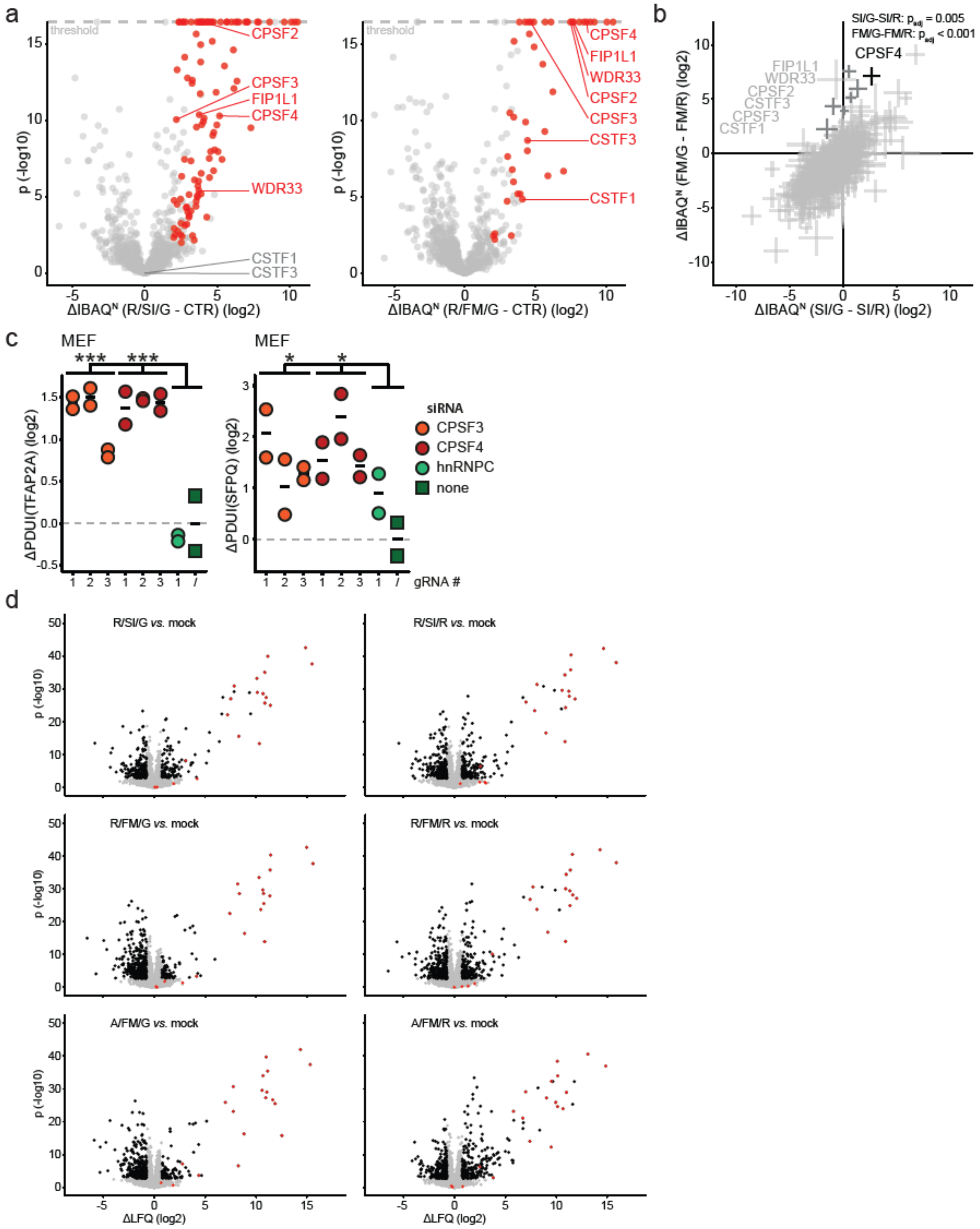

Supplementary Figure 3 | Amino acid positions 103 and 106 dictate the occurrence of host shutoff effect but not APA. (a-b) Related to Fig. 3a – d, statistics are described in the methods section. **(a)** Volcano plots depicting enrichment of proteins in AP-MS of depicted NS1 mutant proteins over control (ThoV M) (y-axis depicts unadjusted p-values). Red dots indicate significance as described in the methods section. CPSF, CSTF and related factors are further highlighted. **(b)** Scatterplot depicting relative enrichment of proteins in comparison between indicated effector domains. CPSF and CSTF complex components, as well as related factors are further highlighted, and the FDR-adjusted p-values for CPSF4 are shown. **(c)** MEFs were transfected with shRNA against Cpsf3 (3 shRNA), Cpsf4 (3 shRNA), Hnrnpc (1 shRNA) or none. 48 hours post-transfection, the cellular RNA was used for RT-qPCR based

quantification of APA of depicted genes. ΔPDUI as a measure of APA status is shown alongside mean for 2 separately processed wells. Statistics were calculated using two-sided Student's t-test as depicted. n.s. $p>0.05$, * $p<0.05$, ** $p<0.01$, *** $p<0.001$. **(d)** Volcano plots showing log2 fold-changes and associated unadjusted p-values between indicated conditions. Statistically significant up- and down-regulated proteins are further highlighted (black) alongside viral proteins (red) (statistics are further described in the methods section).

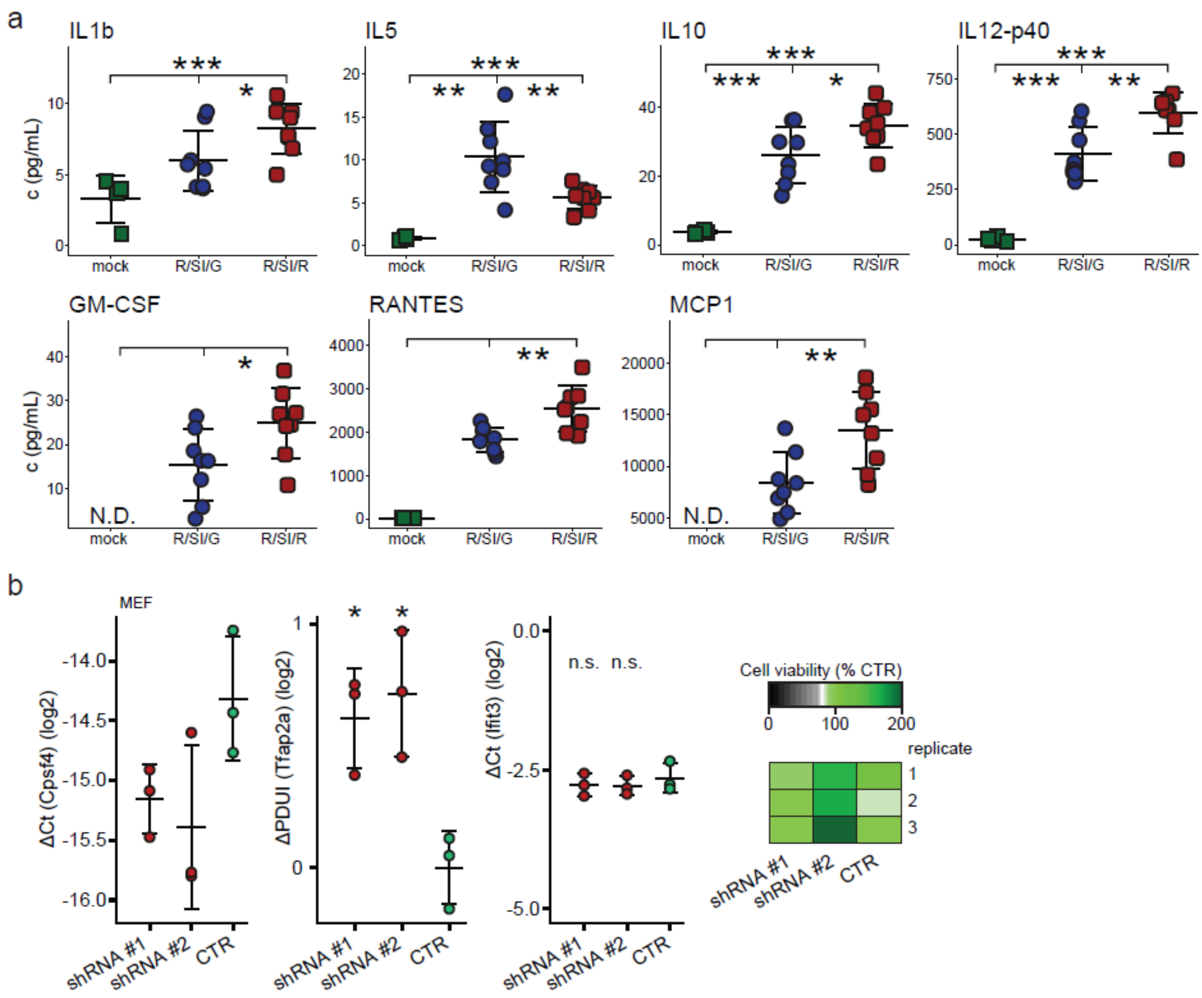

Supplementary Figure 4 | IAV-induced APA of host transcripts inversely correlates with secretion of pro-inflammatory cytokines and interferons. (a) Related to Fig. 5a. C57BL/6J mice were infected with R/SI/G or R/SI/R IAV strains (10e5 pfu, intranasal) or left uninfected. 3 days post infection, secreted cytokines were quantified from BALF (Supp. Data 6). N=8 (4 for mock-infection) shown alongside mean +/- sd. Plots depict significantly changing cytokines between R/SI/G and R/SI/R infected mice. **(b)** Related to Fig. 6c. MEFs (3 independent wells) were transfected with shRNA targeting Cpsf4 or Hnrnpc (CTR) 1 day prior to infection with R/SI/R. 24 hours post infection, *Cpsf4* (left) and *Ifit3* (right) transcript abundance and Tfap2a APA (middle) were quantified by RT-qPCR, shown alongside mean +/- sd. At the time of harvest, cell viability was quantified as described in methods section (far right). ΔCt values were calculated relative to the housekeeping gene *ACTB*. Statistics were calculated using two-sided equal variance t-test. n.s. p>0.05, * p<0.05, ** p<0.01, *** p<0.001.

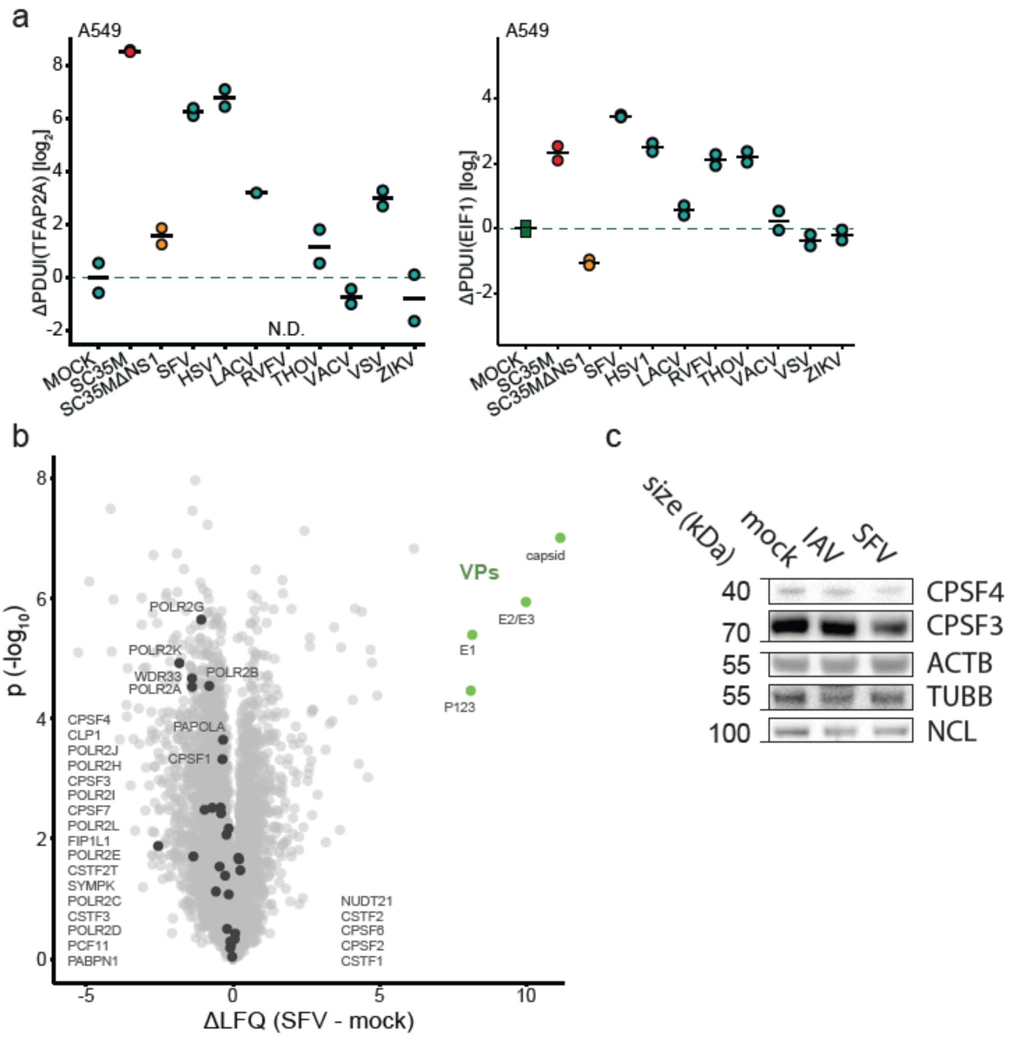

Supplementary Figure 5 | Host transcriptional and in particular polyadenylation machinery is a common target point of pathogenic viruses. (a) Related to Fig. 7a. 2 separate wells of A549 cells were infected with indicated viruses at MOI 1 for 24 hours, followed by quantification of APA of depicted host transcripts (shown alongside mean). **(b)** Related to Fig. 7b. THP-1 cells were left uninfected or infected with SFV at MOI 1 for 24 hours, followed by LC-MS/MS based quantification of protein abundances. Volcano plot depicts protein abundance changes between infected and uninfected conditions alongside unadjusted p-values (two-sided equal variance t-test). Selected transcription-related complex components are highlighted (Supp. Data 7). **(c)** Related to Fig. 7b. A549 cells were infected with SFV at MOI 3 for 24 hours, followed by western blot based detection of depicted protein abundances. The presented data is representative of 2 independent repeats.
