# Supplementary material for: mRNA 3’UTR lengthening by alternative polyadenylation attenuates inflammatory responses and correlates with virulence of Influenza A virus
Source: Nat Commun. 2023 Aug 15;14:4906. doi: 10.1038/s41467-023-40469-6 (PMC10427651; doi:10.1038/s41467-023-40469-6)
Supplement: Supplementary file 2 — Description of additional supplementary files [file 41467_2023_40469_MOESM2_ESM.docx]

**Supplementary data 1**

Title: Results of differential gene expression analysis performed on RNA-seq data from mock- or IAV/PR8-infected cell line A549.

Description: Results of differential gene expression analysis performed on RNA-seq data from mock- or IAV/PR8-infected cell line A549. Gene.name - name of the gene; geneID - Ensembl gene ID (with gene ID version); biotype - gene biotype; baseMean - mean of normalised counts of all samples; log2FoldChange - log2 change between IAV/PR8- and mock-infected condition; p.value and p.adj (Bonf.) - p values and Bonferroni-adjusted p values, respectively. Statistics are further described in the materials and methods.

**Supplementary data 2**

Title: Results of alternative polyadenylation analysis performed on RNA-seq data from mock- or IAV/PR8-infected cell line A549.

Description: Results of alternative polyadenylation analysis performed on RNA-seq data from mock- or IAV/PR8-infected cell line A549. Gene.name - Gene name; ensembl_gene_id - ensembl gene ID; transcript ID - ensembl transcript ID; Chromosome, Strand, Loci - Chromosome, strand and gene locus (hg38); biotype - gene type; p.value - p value of enrichment; p.adjust - FDR-adjusted p value; PD - percentage difference; r - Pearson product moment correlation coefficient; mode - for significant genes either 3'UTR lengthening or shortening; n_predictedAPA - predicted number of polyA sites. Statistics are further described in the materials and methods.

**Supplementary data 3**

Title: Results of the analysis of splicing isoform changes performed on RNA-seq data from mock- or IAV/PR8-infected cell line A549.

Description: Results of the analysis of splicing isoform changes performed on RNA-seq data from mock- or IAV/PR8-infected cell line A549. geneID.version - ensembl gene ID with version; geneName - Gene name; Loci - position of the gene (hg38); Manual.Annotation - Manually annotated association of isoform changes (CDS or 3'UTR); switch.pval - p value; expr.Mock, expr. PR8 and log2FC - expression of the gene in mock-, PR8-infected cells and log2 fold change, respectively; geneType - type of the gene. Statistics are further described in the materials and methods.

**Supplementary data 4**

Title: Results of AP-LC-MS/MS of HA-tagged NS1 and NS1 effector domains.

Description: Results of AP-LC-MS/MS of HA-tagged NS1 and NS1 effector domains. Majority.protein.Ids, external_gene_name - ensembl protein IDs and gene names, associated to individual protein groups, respectively; iBAQ.[sample name].[number] - normalised log2 iBAQ values, where [sample name] is an HA-tagged construct and [number] is the replicate number; RSIG_interactor, RSIG_RBD_dep., RSIG_ED_spec. - RSIG interactors, RSIG RNA-binding domain dependent interactors and RSIG effector domain dependent interactors, respectively; RFMG_interactor, RFMG_RBD_dep., RFMG_ED_spec. - RFMG interactors, RFMG RNA-binding domain dependent interactors and RFMG effector domain dependent interactors, respectively; G184_specific - G184 specific interactors. ntrf and ntrf2 - transfection vehicle only controls. Statistics are further described in the materials and methods.

**Supplementary data 5**

Title: Results of proteome analysis of A549 cells, infected with different PR8-based IAV mutant viruses.

Description: Results of proteome analysis of A549 cells, infected with different PR8-based IAV mutant viruses. Majority.protein.Ids, Gene.name - ensembl protein IDs and gene names, associated to individual protein groups, respectively; [sample name]_[number] - log2 LFQ values, where [sample name] is the identifier of the mutant virus and [number] is the replicate number; [strain], [strain].p, [strain].p.adj (yellow) - log2 fold changes, p values and fdr-adjusted p values associated with comparison of [strain] and mock-infected cells; [RSIX/RFMX/AFMX], [RSIX/RFMX/AFMX].p, [RSIX/RFMX/AFMX].p.adj - log2 fold changes, p values and fdr-adjusted p values associated with comparison between RSIG and RSIR, RFMG and RFMR, and AFMG and AFMR infected cells, respectively. Statistics are further described in the materials and methods.

**Supplementary data 6**

Title: Concentration of cytokines in bronchoalveolar lavage fluid from mice, infected with IAV.

Description: Concentration of cytokines in bronchoalveolar lavage fluid from mice, infected with 50.000 pfu of indicated viruses, 1 and 3 days post infection. Concentrations below detection limit were thresholded to zero. All values are in pg/mL.

**Supplementary data 7**

Title: Results of proteome analysis of THP1 cells, infected with SFV.

Description: Results of proteome analysis of THP1 cells, infected with SFV1. Majority.protein.Ids, Gene.names - Uniprot IDs and gene names, associated to individual protein groups, respectively; [sample name]_[number] - log2 LFQ values, where [sample name] is the identifier of the virus and [number] is the replicate number; SFV log2 fold change, SFV.p - log2 fold changes and p-values associated with comparison of SFV and mock-infected cells (two-sided equal variance t-test).
