# Supplementary material for: mRNA 3’UTR lengthening by alternative polyadenylation attenuates inflammatory responses and correlates with virulence of Influenza A virus
Source: Nat Commun. 2023 Aug 15;14:4906. doi: 10.1038/s41467-023-40469-6 (PMC10427651; doi:10.1038/s41467-023-40469-6)
Supplement: Supplementary file 10 — Reporting Summary [file 41467_2023_40469_MOESM10_ESM.pdf]

nature portfolio

Corresponding author(s): Prof. Dr. Andreas Pichlmair

Last updated by author(s): Jul 12, 2023

Reporting Summary

Nature Portfolio wishes to improve the reproducibility of the work that we publish. This form provides structure for consistency and transparency in reporting. For further information on Nature Portfolio policies, see our Editorial Policies and the Editorial Policy Checklist.

Statistics

For all statistical analyses, confirm that the following items are present in the figure legend, table legend, main text, or Methods section.

n/a	Confirmed	
☐	☒	The exact sample size (*n*) for each experimental group/condition, given as a discrete number and unit of measurement
☐	☒	A statement on whether measurements were taken from distinct samples or whether the same sample was measured repeatedly
☐	☒	The statistical test(s) used AND whether they are one- or two-sided<br>*Only common tests should be described solely by name; describe more complex techniques in the Methods section.*
☐	☒	A description of all covariates tested
☐	☒	A description of any assumptions or corrections, such as tests of normality and adjustment for multiple comparisons
☐	☒	A full description of the statistical parameters including central tendency (e.g. means) or other basic estimates (e.g. regression coefficient) AND variation (e.g. standard deviation) or associated estimates of uncertainty (e.g. confidence intervals)
☐	☒	For null hypothesis testing, the test statistic (e.g. *F*, *t*, *r*) with confidence intervals, effect sizes, degrees of freedom and *P* value noted<br>*Give P values as exact values whenever suitable.*
☒	☐	For Bayesian analysis, information on the choice of priors and Markov chain Monte Carlo settings
☐	☒	For hierarchical and complex designs, identification of the appropriate level for tests and full reporting of outcomes
☐	☒	Estimates of effect sizes (e.g. Cohen's *d*, Pearson's *r*), indicating how they were calculated

Our web collection on statistics for biologists contains articles on many of the points above.

Software and code

Policy information about availability of computer code

Data collection	MaxQuant version 1.5.6.2, QuantStudio Design and Analysis Software 1.4.3
Data analysis	R version 4.0.2, bedtools 2.26.0, APAtrap 1.0, DESeq2 1.36, SALMON 0.13.1, IsoformSwitchAnalyzeR 1.8

For manuscripts utilizing custom algorithms or software that are central to the research but not yet described in published literature, software must be made available to editors and reviewers. We strongly encourage code deposition in a community repository (e.g. GitHub). See the Nature Portfolio guidelines for submitting code & software for further information.

Data

Policy information about availability of data

All manuscripts must include a data availability statement. This statement should provide the following information, where applicable:

- Accession codes, unique identifiers, or web links for publicly available datasets
- A description of any restrictions on data availability
- For clinical datasets or third party data, please ensure that the statement adheres to our policy

The mass spectrometry proteomics data have been deposited to the ProteomeXchange Consortium via the PRIDE88 partner repository with the dataset identifier PXD040456 (reviewer username: reviewer_pxd040456@ebi.ac.uk and password: Cp54DGN8). Sequencing data is publically available via ENA repository (PRJEB54734). Source data are provided with this paper.

Research involving human participants, their data, or biological material

Policy information about studies with human participants or human data. See also policy information about sex, gender (identity/presentation), and sexual orientation and race, ethnicity and racism.

Reporting on sex and gender	N/A
Reporting on race, ethnicity, or other socially relevant groupings	N/A
Population characteristics	N/A
Recruitment	N/A
Ethics oversight	N/A

Note that full information on the approval of the study protocol must also be provided in the manuscript.

Field-specific reporting

Please select the one below that is the best fit for your research. If you are not sure, read the appropriate sections before making your selection.

☒ Life sciences ☐ Behavioural & social sciences ☐ Ecological, evolutionary & environmental sciences

For a reference copy of the document with all sections, see nature.com/documents/nr-reporting-summary-flat.pdf

Life sciences study design

All studies must disclose on these points even when the disclosure is negative.

Sample size	No sample-size pre-calculations were performed - sample sizes were chosen based on prior experience.
Data exclusions	No data was excluded
Replication	All described data was replicated independently at least one additional time
Randomization	No randomization was done, not applicable to this study.
Blinding	No blinding was used, we considered it not required for this study.

Reporting for specific materials, systems and methods

We require information from authors about some types of materials, experimental systems and methods used in many studies. Here, indicate whether each material, system or method listed is relevant to your study. If you are not sure if a list item applies to your research, read the appropriate section before selecting a response.

Materials & experimental systems

n/a	Involved in the study	
☐	☒	Antibodies
☐	☒	Eukaryotic cell lines
☒	☐	Palaeontology and archaeology
☐	☒	Animals and other organisms
☒	☐	Clinical data
☒	☐	Dual use research of concern
☒	☐	Plants

Methods

n/a	Involved in the study	
☒	☐	ChIP-seq
☒	☐	Flow cytometry
☒	☐	MRI-based neuroimaging

Antibodies

Antibodies used	the following antibodies were used: GFP (Invitrogen, A6455), HA (Cell Signaling, 2367), HIST3H3 (Abcam, ab1791-100), CPSF4 (Abcam, ab131218), CPSF3 (Abcam, ab72295), ACTB-HRP (Santa Cruz, sc-47778), TUBB (Santa Cruz, sc-9104), NCL (Thermo Fisher Scientific, 39-6400), msHRP (Cell Signaling, 7076) and rbHRP (Cell Signaling, 7074)

Validation | Validation was performed by the supplier - the data is available on the respective websites.

Eukaryotic cell lines

Policy information about cell lines and Sex and Gender in Research

Cell line source(s)	Cell lines were obtained from ATCC (HEK293T, HeLa, A549, Vero E6)
Authentication	Authentication was performed by STR profiling
Mycoplasma contamination	All cell lines were tested and shown to be mycoplasma-free.
Commonly misidentified lines (See ICLAC register)	not used

Animals and other research organisms

Policy information about studies involving animals; ARRIVE guidelines recommended for reporting animal research, and Sex and Gender in Research

Laboratory animals	8 weeks-old female C57BL/6J mice were purchased from Janvier Labs
Wild animals	not used
Reporting on sex	Not considered
Field-collected samples	not used
Ethics oversight	All experiments with mice were carried out in accordance with the guidelines of the Federation for Laboratory Animal Science Associations (FELASA) and the national animal welfare body. Experiments were in compliance with the German animal protection law and were approved by the animal welfare committee of the Regierungspräsidium Freiburg (permits X-18/01K and G-15/125).

Note that full information on the approval of the study protocol must also be provided in the manuscript.
