# Supplementary figures and images for: mRNA 3’UTR lengthening by alternative polyadenylation attenuates inflammatory responses and correlates with virulence of Influenza A virus
Source: Nat Commun. 2023 Aug 15;14:4906. doi: 10.1038/s41467-023-40469-6 (PMC10427651; doi:10.1038/s41467-023-40469-6)

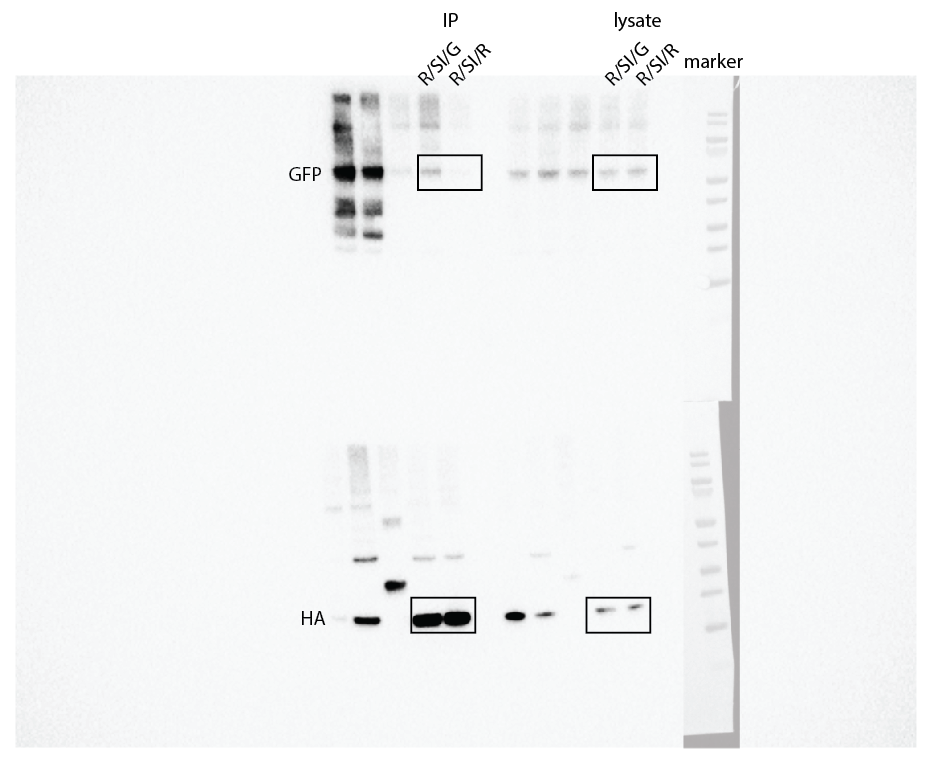

Supplement: Supplementary file 12 — Source Data [file 41467_2023_40469_MOESM12_ESM.zip › F3d_WBs.png]

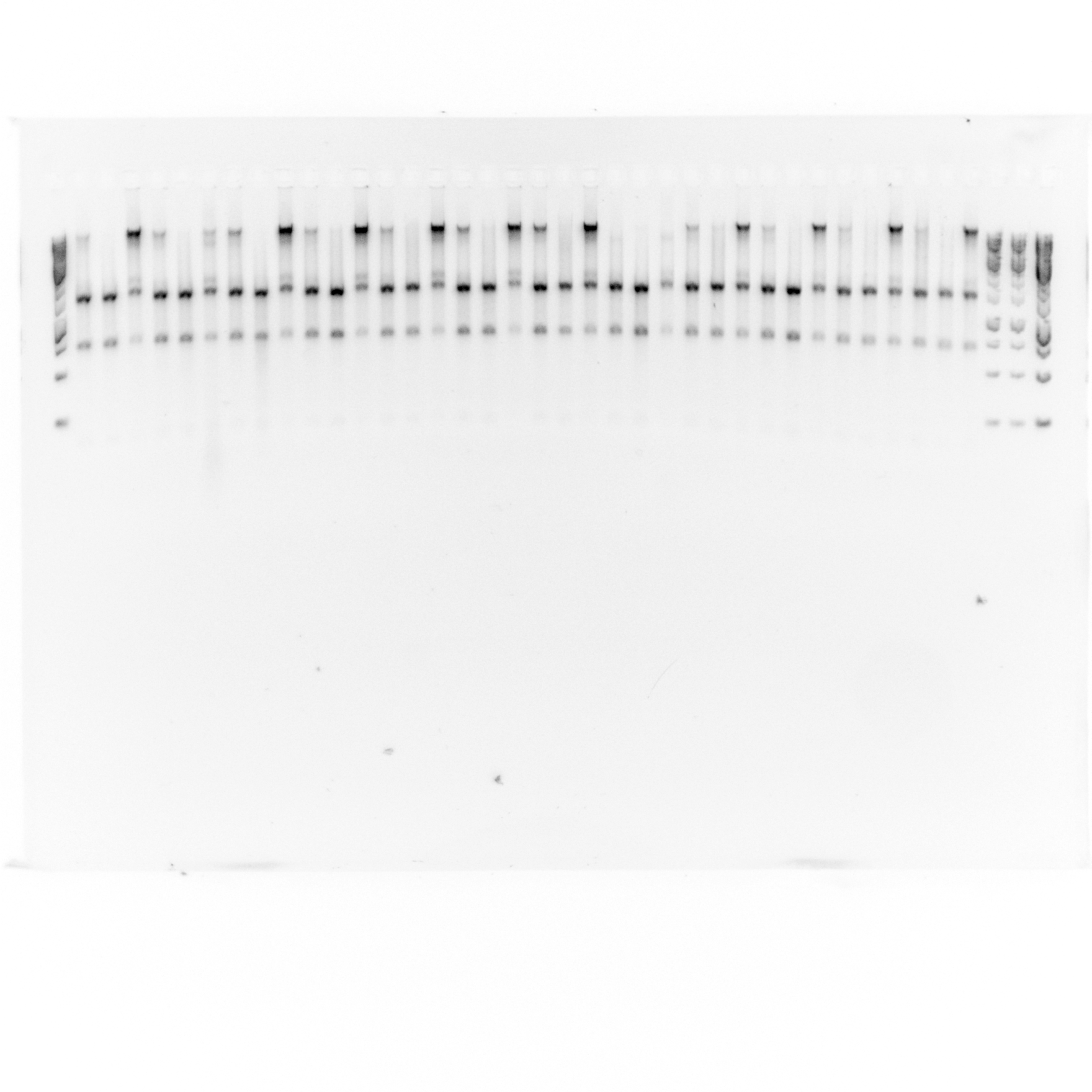

Supplement: Supplementary file 12 — Source Data [file 41467_2023_40469_MOESM12_ESM.zip › SF2c_Agarose_gel_inverted.png]

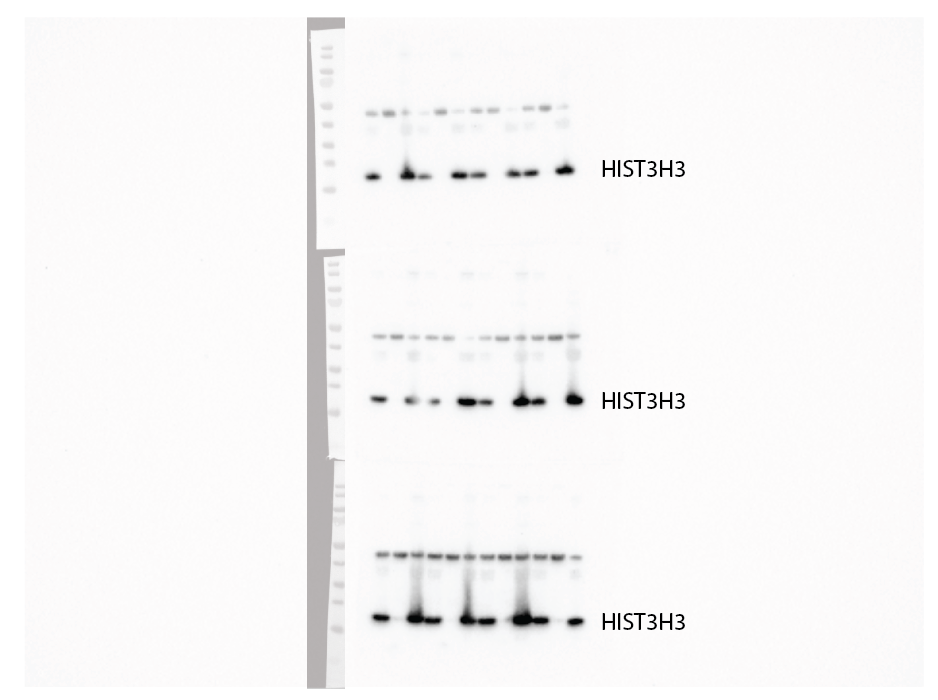

Supplement: Supplementary file 12 — Source Data [file 41467_2023_40469_MOESM12_ESM.zip › SF2c_WBs.png]

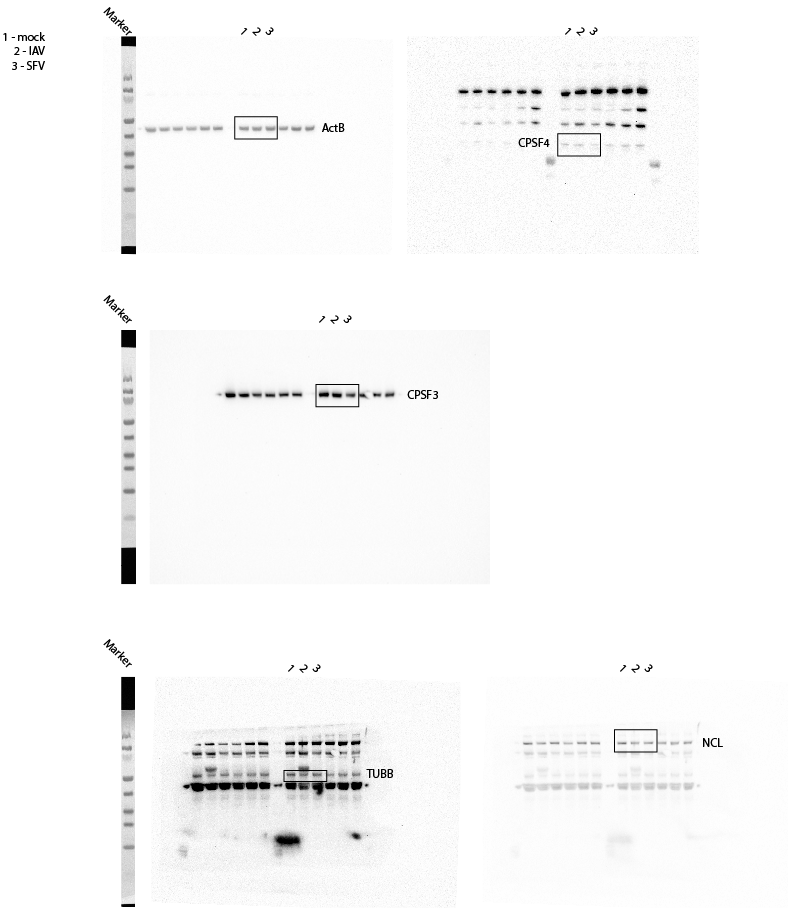

Supplement: Supplementary file 12 — Source Data [file 41467_2023_40469_MOESM12_ESM.zip › SF5c_WBs.png]
